# Development of an Assessment Tool for Social Cognition in Older Adults

**DOI:** 10.1002/alz70857_103012

**Published:** 2025-12-25

**Authors:** Beatriz Figueiredo e Silva, Luiz Alves Ferreira Júnior, Jéssica Diniz Ferreira, Amanda Pelinson Gomes Tiago, Júlia Costa/C Dias, Marcela Sena Braga, Debora Marques de Miranda, Marco Aurélio Romano‐Silva, Maria Aparecida Camargos Bicalho, Jonas Jardim de Paula, Bernardo de Mattos Viana

**Affiliations:** ^1^ Universidade Federal de Minas Gerais, Belo Horizonte, Minas Gerais, Brazil; ^2^ Older Adult's Psychiatry and Psychology Extension Program (PROEPSI), School of Medicine, Universidade Federal de Minas Gerais (UFMG), Belo Horizonte, Minas Gerais, Brazil; ^3^ Molecular Medicine Postgraduate Program, School of Medicine, Universidade Federal de Minas Gerais (UFMG), Belo Horizonte, Minas Gerais, Brazil; ^4^ Cog‐Aging Research Group, Universidade Federal de Minas Gerais (UFMG), Belo Horizonte, Minas Gerais, Brazil; ^5^ Neurotec R National Institute of Science and Technology (INCT‐Neurotec R), Faculty of Medicine, Universidade Federal de Minas Gerais (UFMG), Belo Horizonte, Minas Gerais, Brazil; ^6^ Department of Psychiatry, School of Medicine, Federal University of Minas Gerais, Belo Horizonte, Minas Gerais, Brazil; ^7^ Geriatrics and Gerontology Center Clinical Hospital of University of Minas Gerais, Belo Horizonte, Minas Gerais, Brazil

## Abstract

**Background:**

Social cognition refers to the set of mental processes involved in the interpretation of emotional and social information that enables individuals to respond appropriately to others' intentions and behaviors. In Brazil, there is a scarcity of assessment tools for evaluating social cognition in older adults, particularly developed for this population.

**Objective:**

(1) To develop and adapt an assessment tool for social cognition for older adults; (2) To investigate content validity evidence for this instrument; (3) To apply the instrument in a pilot group.

**Methods:**

(1) The instrument was developed based on the Theory of Mind (ToM) tasks, including False Belief tasks, Strange Stories, and Faux‐Pas tasks. (2) The instrument underwent expert review, with judges selected based on their expertise: 3 psychologists/neuropsychologists, 1 psychogeriatrician, and 2 geriatricians. The judges evaluated the instrument's items in terms of comprehensiveness, clarity, and relevance. Each item of the instrument was quantified using the Content Validity Index (CVI). (3) After incorporating the judges' suggestions, the instrument was administered to a pilot group of 10 participants.

**Results:**

The preliminary version of the social cognition assessment tool comprises of 12 subtests: 4 assessing first‐ and second‐order false beliefs, 4 evaluating pragmatic inference (Strange Stories task), and 4 measuring faux‐pas. The overall agreement among judges ranged from 0.66 to 1.0 (66% to 100%), with most tasks achieving over 80% agreement. Tasks that achieved less than 80% were reviewed and reassessed by the judges. The pilot study included 10 older adults with a mean age of 67.9 years (SD = 7.0) and a mean education level of 10.7 years (SD = 3.6). Overall, participants demonstrated good task comprehension, but some items required modifications to enhance clarity for older adults. Participants performed better on false belief and Strange Stories tasks (cognitive processes), but had greater difficulty with faux‐pas tasks (both cognitive and affective processes).

**Conclusion:**

This instrument has been developed especially considering Brazilian older adults and their background. Nevertheless, modifications were needed to enhance clarity and comprehension. The next phase is to assess representative sample of older adults with and without clinical syndromes for standardization and to create the norms.

